# Supplementary material for: Bacteriophage defends murine gut from Escherichia coli invasion via mucosal adherence
Source: Nat Commun. 2024 Jun 4;15:4764. doi: 10.1038/s41467-024-48560-2 (PMC11150434; doi:10.1038/s41467-024-48560-2)
Supplement: Supplementary file 1 — Supplementary Information [file 41467_2024_48560_MOESM1_ESM.pdf]

Supplemental information

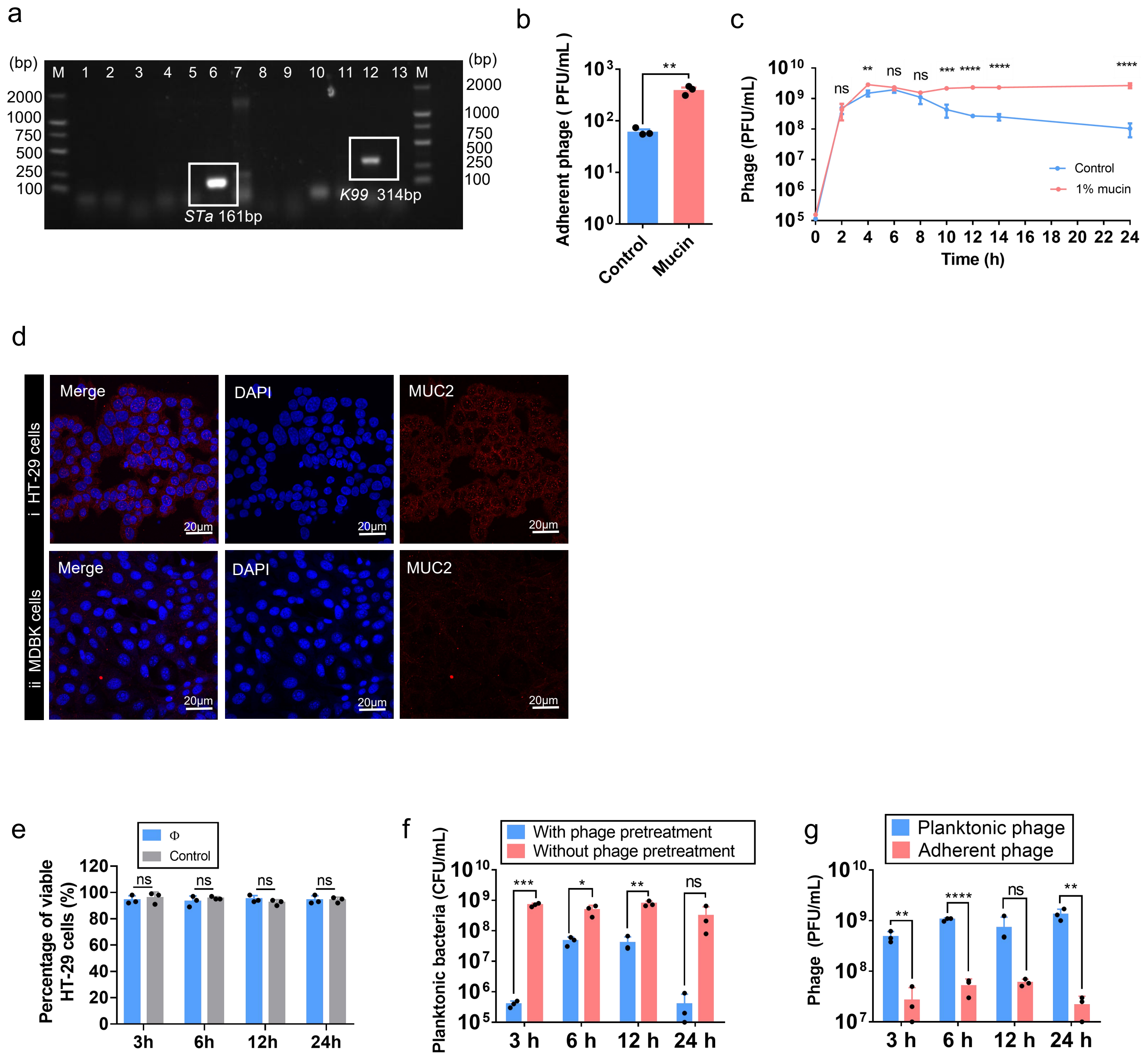

Supplemental information

Supplementary Fig 1. (a) Detection of virulence genes of SH232 by PCR. Line 1~13, *stx1*, *stx2*, *aggR*, *astA*, *hly*, *STa*, *STb*, *LT*, *bfpB*, *eae*, *invE*, *K99*, *ipah;* M, Marker. (b) The number of phage adhere to LB agar plates coated with or without mucin. (c) The titer of øPNJ-6 during 24 h when growing in LB medium with or without 1% mucin. (d) Fluorescence microscope photograph of MUC2 expression levels in HT-29 cells or in MDBK cells (×100); Red indicates MUC2, and blue indicates cell nucleus. (e) HT-29 cells were incubated with øPNJ-6 lysate and the viability of HT-29 cells was measured by time point. (f) The number of ETEC in the supernatant of HT-29 cells, at different time points (3h, 6 h, 12 h, and 24 h). (g)The number of phage in the supernatant and phage adhering to the cells *in vitro*. Data are presented as mean values +/− SD. n = 3 biologically independent experiments. *P*-values are calculated by Multiple t test one per-row (Supplementary information Fig. 1b, 1e, 1f, 1g) or Two-way ANOVA (Supplementary information Fig. 1c) (*, *P* ≤0.05; **, *P* ≤0.01; ***, *P*≤0.001; ****, *P* ≤ 0.0001). Source data are provided as a Source Data file.

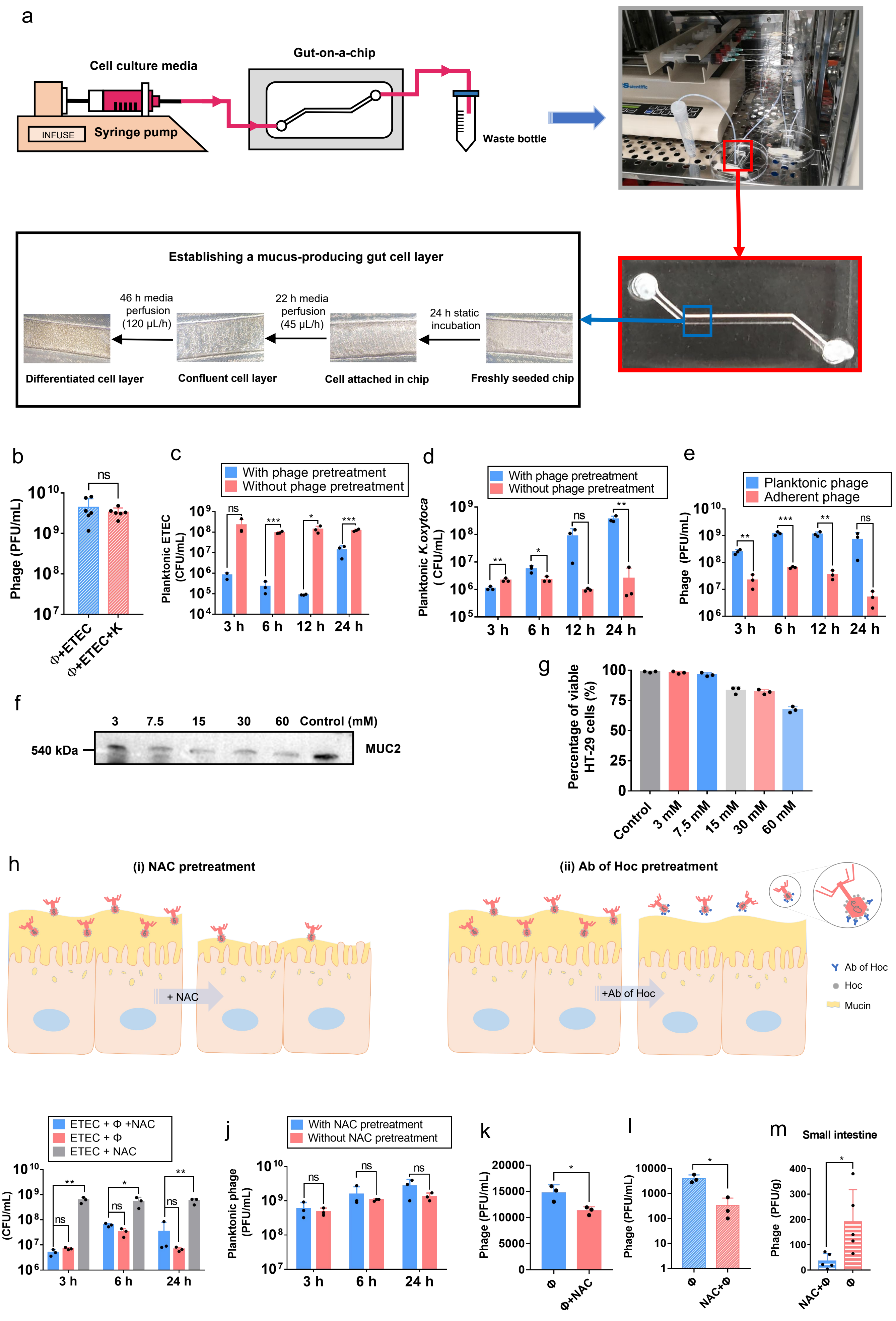

a
Cell culture media
INFUSE
Syringe pump
Gut-on-a-chip
Waste bottle
Establishing a mucus-producing gut cell layer
46 h media perfusion (120 μL/h)
22 h media perfusion (45 μL/h)
24 h static incubation
Differentiated cell layer
Confluent cell layer
Cell attached in chip
Freshly seeded chip
b
Phage (PFU/mL)
Φ+ETEC
Φ+ETEC+K
c
With phage pretreatment
Without phage pretreatment
Planktonic ETEC (CFU/mL)
d
Planktonic K.oxytoca (CFU/mL)
e
Planktonic phage
Adherent phage
Phage (PFU/mL)
f
3 7.5 15 30 60 Control (mM)
540 kDa
MUC2
g
Percentage of viable HT-29 cells (%)
Control
3 mM
7.5 mM
15 mM
30 mM
60 mM
h
(i) NAC pretreatment
+ NAC
(ii) Ab of Hoc pretreatment
+Ab of Hoc
Ab of Hoc
Hoc
Mucin
i
ETEC + Φ +NAC
ETEC + Φ
ETEC + NAC
Planktonic ETEC (CFU/mL)
j
With NAC pretreatment
Without NAC pretreatment
Planktonic phage (PFU/mL)
k
Phage (PFU/mL)
Φ
Φ+NAC
l
Phage (PFU/mL)
Φ
NAC+Φ
m
Small intestine
Phage (PFU/g)
NAC+Φ
Φ

Supplementary Fig 2. (a) The schematic diagram of gut-on-a-chip. The image was created by PowerPoint. (b) The phage number in gut-on-a-chip model with ETEC or ETEC + *K.oxytoca.* The number of ETEC (c) and *K.oxytoca* (d) in the supernatant, and the phage number (e) in the competitive model with ETEC and *K.oxytoca* at different time points (3 h, 6 h, 12 h, and 24 h). (f)The removal of MUC2 of HT-29 when treated with different concentrations of NAC (3 mM, 7.5 mM, 15 mM, 30 mM, and 60 mM). (g) The proportion of viable HT-29 cells when treated with different NAC concentrations. (h-i) The sketch map showing the effect of NAC treatment on the interaction between øPNJ-6 and the intestinal tract; NAC treatment resulted in a reduction of mucus in goblet cells, which subsequently led to a decrease in the number of adherent phage; (h-ii) The sketch map showing the effect of antibody blocking to Hoc on the interaction between øPNJ-6 and the intestinal tract. After Hoc antibody pretreatment, øPNJ-6 lost the ability to adhere to mucus. The image was created by PowerPoint. The number of ETEC (i) and phage (j) in the supernatant *in vitro* with or without NAC treatment at different time points (3 h, 6 h, 24 h). (k) In the absence of ETEC, the øPNJ-6 number in groups with or without NAC pretreatment *in vitro*. (l) In the absence of ETEC, the øPNJ-6 number in groups with or without NAC pretreatment in the gut-on-a-chip. (m) The number of phage that adhere to the small intestine of mice pre-treated with or without NAC. Data are presented as mean values +/− SD. n = 3 biologically independent experiments. *P*-values are calculated by Multiple t test one per-row (*, $P \leq 0.05$; **, $P \leq 0.01$; ***, $P \leq 0.001$; ****, $P \leq 0.0001$). Source data are provided as a Source Data file.

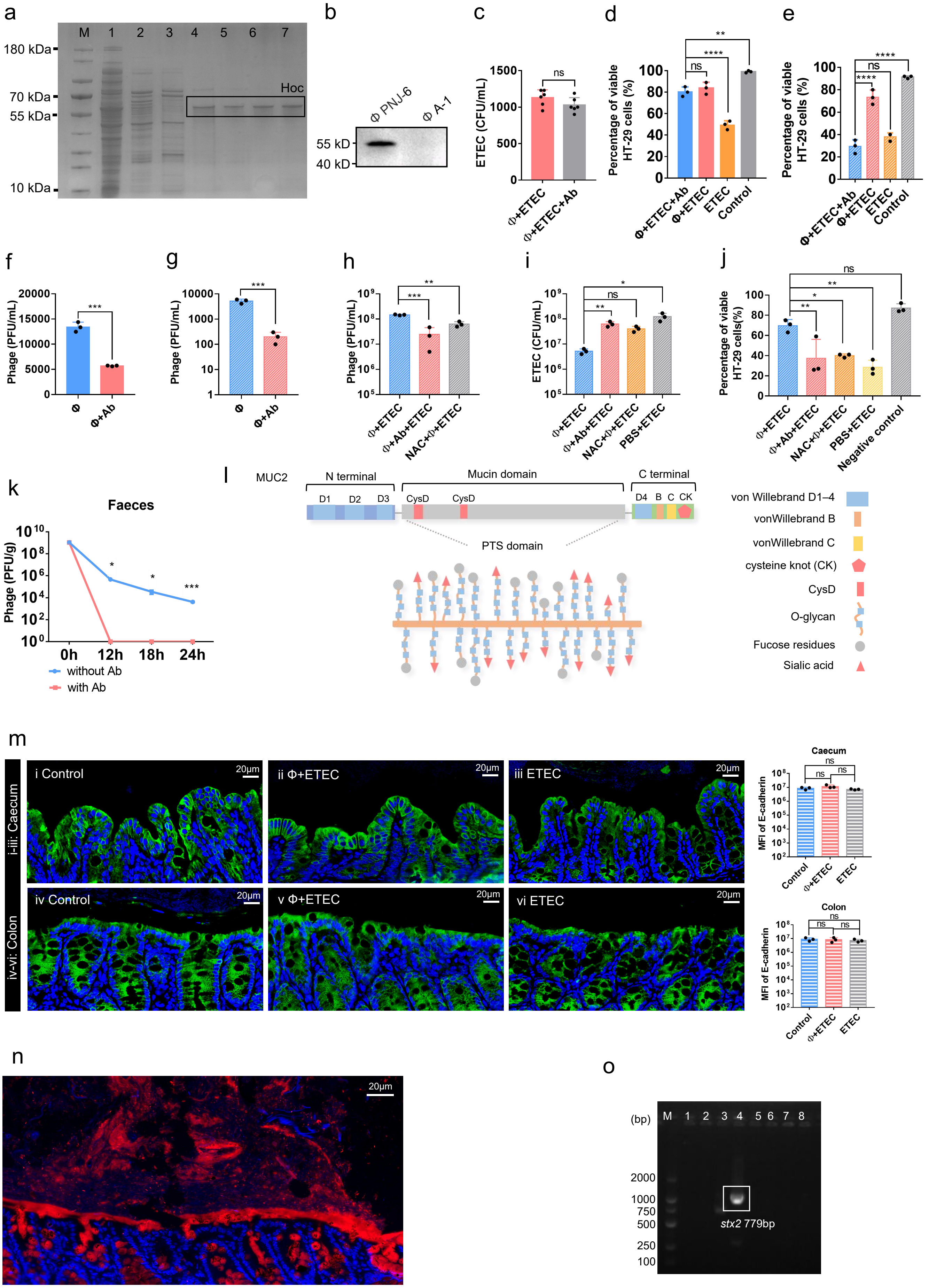

a
M 1 2 3 4 5 6 7
180 kDa
70 kDa
55 kDa
10 kDa
Hoc
b
Φ PNJ-6
Φ A-1
55 kD
40 kD
c
ETEC (CFU/mL)
ns
Φ+ETEC
Φ+ETEC+Ab
d
Percentage of viable HT-29 cells (%)
Φ+ETEC+Ab
Φ+ETEC
ETEC
Control
e
Percentage of viable HT-29 cells (%)
Φ+ETEC+Ab
Φ+ETEC
ETEC
Control
f
Phage (PFU/mL)
Φ
Φ+Ab
g
Phage (PFU/mL)
Φ
Φ+Ab
h
Phage (PFU/mL)
Φ+ETEC
Φ+Ab+ETEC
NAC+Φ+ETEC
i
ETEC (CFU/mL)
Φ+ETEC
Φ+Ab+ETEC
NAC+Φ+ETEC
PBS+ETEC
j
Percentage of viable HT-29 cells(%)
Φ+ETEC
Φ+Ab+ETEC
NAC+Φ+ETEC
PBS+ETEC
Negative control
k
Faeces
Phage (PFU/g)
0h 12h 18h 24h
without Ab
with Ab
l
MUC2
N terminal
Mucin domain
C terminal
D1 D2 D3
CysD CysD
D4 B C CK
PTS domain
von Willebrand D1–4
vonWillebrand B
vonWillebrand C
cysteine knot (CK)
CysD
O-glycan
Fucose residues
Sialic acid
m
i-iii: Caecum
iv-vi: Colon
i Control
ii Φ+ETEC
iii ETEC
iv Control
v Φ+ETEC
vi ETEC
20μm
Caecum
Colon
MFI of E-cadherin
Control
Φ+ETEC
ETEC
n
20μm
o
(bp)
M 1 2 3 4 5 6 7 8
2000
1000
750
500
250
100
stx2 779bp

Supplementary Fig 3. (a) SDS-PAGE of Hoc protein. (b) Polyclonal antibody against Hoc was used to detect phage øPNJ-6 and phage øA-1 by WB, phage øA-1 designed as the negative control. (c) The lysis ability of phage øPNJ-6 with or without Hoc antibody incubation *in vitro*. (d) The proportion of viable HT-29 cells pre-treated with phage or Hoc antibody-blocked phage *in vitro*. (e) The proportion of viable HT-29 cells pre-treated with phage or Hoc antibody-blocked phage in gut-on-a-chip. (f) In the absence of ETEC, the øPNJ-6 number in groups with or without Hoc antibody pretreatment *in vitro*. (g) In the absence of ETEC, the øPNJ-6 number in groups with or without antibody-coated pretreatment in the gut-on-a-chip. In the pump-paused model, øPNJ-6 titers (h), ETEC SH232 loads (i), and the cell viability (j) in the gut-on-a-chip with or without NAC or Hoc antibody-coated. (k) The number of phage in the feces of mice pre-treated with phage or Hoc antibody-blocked phage. (l) MUC2 undergoes N- and O-glycosylation modifications. The image was created by PowerPoint. (m) Fluorescence microscope photograph of E-cadherin expression levels to confirm the tight junctions in the intestinal epithelium (×60); The bar represents MFI of E-cadherin. Green indicates E-cadherin, and blue indicates cell nucleus. Scale bars, 20 μm. (n) Fluorescence microscope photograph of MUC2 expression levels in the intestinal epithelium (×60). Red indicates MUC2, and blue indicates cell nucleus. Scale bars, 20 μm. (o) The detection of STEC virulence genes by PCR. Line 1~8, *aggR*, *LT*, *stx1*, *stx2*, *bfpB*, *STa*, *STb*, *invE*. M, Marker. Data are presented as mean values +/− SD. n = 3 biologically independent experiments. *P*-values are calculated by unpair t test (Supplementary information Fig. 3c, 3f, 3g, 3m), Multiple t test-one per row (Supplementary information Fig. 3k) or One-way ANOVA (Supplementary information Fig. 3d, 3e, 3h, 3i, 3j) (*, $P \leq 0.05$; **, $P \leq 0.01$; ***, $P \leq 0.001$; ****, $P \leq 0.0001$). Source data are provided as a Source Data file.

Supplementary Table 1. List of primer sequence in this study.

Primer	Sequence	Source
6-*hoc*-F	CAGCAAATGGGTCGCGGATCCATGGCTTTTACAGTTGATATAACTCCTAA	This study
6-*hoc*-R	GTGGTGGTGGTGGTGCTCGAGTTATGGATAGGTATAGATGATACCAGTTTC	This study
6-D1-*hoc*-F	CAGCAAATGGGTCGCGGATCCATGGCTTTTACAGTTGATATAACTCCTAA	This study
6-D1-*hoc*-R	GTGGTGGTGGTGGTGCTCGAGGGTAGTTGTCTGTGTTTTGTTTTGAA	This study
6-D2-*hoc*-F	CAGCAAATGGGTCGCGGATCCCAAAACAAAACACAGACAACTACCTTG	This study
6-D2-*hoc*-R	GTGGTGGTGGTGGTGCTCGAGTGGATTCATTGTCTTCTTATTAACTGTC	This study
6-D3-*hoc*-F	GCTGATATCGGATCCGAATTCAATAAGAAGACAATGAATCCACAGGT	This study
6-D3-*hoc*-R	GTGGTGGTGGTGGTGCTCGAGAAGAGGATGAACATAAGGTAGTTCACC	This study
hoc (T19S-Q21H)-F	GATGATAGTAATCATTTTACTGCTACTCCCAGTGGTGAA	This study
hoc (T19S-Q21H)-R	AAAATGATTACTATCATCTATAACTCCAGTAGGTGTTTTAGGAGT	This study
hoc (E29D-G33V)-F	CAGCAAATGGGTCGCGGATCCATGGCTTTTACAGTTGATATAACTCCTAAAACACCTACTGGAGTTATAGATGAGACTAAGCAGTTTACTGCTACTCCCAGTGGTGATATTGCAGCTGTAACT	This study
hoc (E29D-G33V)-R	TTGTCGACGGAGCTCGAATTCTTATGGATAGGTATAGATGATACCAGTTTC	This study
hoc (T30I-G32A)-F	GTGAAATTGCAGCTGGAACTATTACCTATGCGTGGAGTG	This study
hoc (T30I-G32A)-R	TCCAGCTGCAATTTCACCACTGGGAGTAGCAGTAAA	This study
hoc (V15D-E18G)-F	CAGCAAATGGGTCGCGGATCCATGGCTTTTACAGTTGATATAACTCCTAAAACACCTACTGGAGATAGAGTTGGA	This study
hoc (V15D-E18G)-R	GTGGTGGTGGTGGTGCTCGAGTTATGGATAGGTATAGATGATACCAGTTTC	This study

Primer	Sequence	Source
hoc (E29D)-F	AGTGGTGATACTGGAGGTGGAA CTATTACCTATGC	This study
hoc (E29D)-R	CCTCCAGTATCACCACTGGGAG TAGCAGTAAAC	This study
hoc (G33V)-F	GAAACTGGAGGTGTAACTATTAC CTATGCGTGGAGTGTAGA	This study
hoc (G33V)-R	GTTACACCTCCAGTTTCACCACT GGGAGTAGC	This study
6-*soc*-F	GCTGATATCGGATCCGAATTCAT GGGTGGTTATGTAAACATCAAAA	This study
6-*soc*-R	GTGGTGGTGGTGGTGCTCGAGA CCGCTTACCGGTGTAGGGG	This study
MUC2 - F qPCR	TACCACGACTGGGTCATCTTCA	This study
MUC2 - R qPCR	TGTCTCCGTATGTGCCGTTGTA	This study
GAPDH -F qPCR	CTGGAGAAACCTGCCAAGTA	This study
GAPDH -R qPCR	CTGTTGCTGTAGCCGTATTC	This study
STa-F	TCCCCTCTTTTAGTCAGTCAACT G	[55]
STa-R	GCACAGGCAGGATTACAACAA AGT	[55]
STb-F	GCAATAAGGTTGAGGTGAT	[56]
STb-R	GCCTGCAGTGAGAAATGG AC	[56]
LT-F	TTACGGCGTTACTATCCTCTCTA	[57]
LT-R	GGTCTCGGTCAGATATGTGATTC	[57]
K99-F	TATTATCTTAGGTGGTATGG	This study
K99-R	GGTATCCTTTAGCAGCAGTATT	This study
stx1-F	CGATGTTACGGTTTGTTACTGTG ACAGC	[54]
stx1-R	AATGCCACGCTTCCCAGAATTG	[54]
stx2-F	C CATGACAACGGACAGCAGT T	[54]
stx2-R	CCTGTCAACTGAGCAGCACTTT G	[54]
bfpB-F	GACACCTCATTGCTGAAGTCG	[54]
bfpB-R	CCAGAACACCTCCGTTATGC	[54]
invE-F	CGATAGATGGCGAGAAATTATAT CCCG	[54]
invE-R	CGATCAAGAATCCCTAACAGAAG AATCAC	[54]
aggR-F	ACGCAGAGTTGCCTGATAAAG	[54]
aggR-R	AATACAGAATCGTCAGCATCAGC	[54]
astA-F	TGCCATCAACACAGTATATCCG	[54]
astA-R	ACGGCTTTGTAGTCCTTCCAT	[54]
eae-F	TCAATGCAGTTCCGTTATCAGTT	[54]
eae-R	GTAAAGTCCGTTACCCCAACCTG	[54]
hly-F	ACGATGTGGTTTATTCTGGA	[58]
hly-R	CTTCACGTGACCATACATAT	[58]

Primer	Sequence	Source
ipah-F	CTCGGCACGTTTTAATAGTCTGG	[59]
ipah-R	GTGGAGAGCTGAAGTTTCTCTG C	[59]

Supplemental Table 2. Sources of information on ten *E.coli* strains.

Number	*E.coli* Strain	Time	Location	Origin	Type of Sample
1	014	2023	China	Human	puncture fluid
2	015	2023	China	Human	urine
3	016	2023	China	Human	urine
4	017	2023	China	Human	urine
5	018	2023	China	Human	urine
6	019	2023	China	Human	urine
7	029	2023	China	Human	urine
8	039	2023	China	Human	urine
9	042	2023	China	Human	urine
10	044	2023	China	Human	sputum
